# The Nuclear Localization of the DnaJ-Like Zinc Finger Domain-Containing Protein EDA3 Affects Seed Development in *Arabidopsis thaliana*

**DOI:** 10.3390/ijms21217979

**Published:** 2020-10-27

**Authors:** Meng-Juan Kong, Na Huang, Si-Ming Chen, Han-Yu Liang, Xin-Ya Liu, Zhong Zhuang, Shan Lu

**Affiliations:** State Key Laboratory of Pharmaceutical Biotechnology, School of Life Sciences, Nanjing University, Nanjing 210023, China; 13260820993@163.com (M.-J.K.); DZ1930003@smail.nju.edu.cn (N.H.); 15221535226@163.com (S.-M.C.); MG1930011@smail.nju.edu.cn (H.-Y.L.); MG1930013@smail.nju.edu.cn (X.-Y.L.); njcs2006@126.com (Z.Z.)

**Keywords:** chloroplast, DnaJ-like, EDA3, female gametophyte, nucleus, PSA2, zinc finger domain

## Abstract

The DnaJ-like zinc finger domain-containing proteins are involved in different aspects of plastid function and development. Some of these proteins were recently reported to have dual subcellular localization in the nucleus and plastids. One member of this family, PSA2 (AT2G34860), was found to localize to the thylakoid lumen and regulate the assembly of photosystem I (PSI). However, PSA2 was also annotated as Embryo sac Development Arrest 3 (EDA3) from the observation that its embryo sac development was arrested at the two-nuclear stage. In this study, we characterized the *eda3* mutant, and demonstrated that, as compared with the wild-type (WT) plants, the mutant has shorter siliques, fewer siliques per plant, and fewer seeds per silique. Both aborted and undeveloped ovules were observed in siliques of the mutant. By immunoblot analysis, we found that, different from the chloroplast localization in mature leaves, EDA3 localizes in the nucleus in seeds. A nuclear localization signal was identified from the deduced amino acid sequence of EDA3, and also proved to be sufficient for directing its fusion peptide into the nucleus.

## 1. Introduction

The alternation of generations between sporophyte and gametophyte builds a complete life cycle of flowering plants, which is a joint effort of a broad spectrum of genes, such as those for gametogenesis, seed development, and subsequent growth. Although various metabolic and developmental scenarios, including pigment biosynthesis and flower development, are involved in this process, genes required for completing the life cycle under normal conditions are crucial for plants [1]. Different approaches have been conducted to determine these genes, among which those for embryo development have received particular attention [2,3]. For example, a comprehensive screening of Ds transposon insertion lines of *Arabidopsis thaliana* identified 130 genes that conferred defects in female gametophyte development and function [4]. Some of these mutants have defective mitoses during embryo sac development, exhibit an abnormal number or position of nuclei, or fail to fuse polar nuclei and become multicellular blastocysts. Among these mutants, 41 were termed as *Embryo sac Development Arrest* (*EDA*) genes for their defects in embryo sac development at various stages [4]. However, this study was mainly based on phenotype observation, and only a few of the EDA members were subsequently characterized, such as EDA17, a glucose-methanol-choline oxidoreductase for anther cutin biosynthesis, and EDA13, a WD40 protein involved in 18S rRNA biogenesis for gametogenesis [5,6].

Among these *EDA* genes, an interruption of *EDA3* (also known as *PSA2*, *At2g34860*) demonstrated an arrested development of embryo sacs at the two-nuclear stage during megagametogenesis [4]. Sequence analysis revealed that EDA3 is a member of the DnaJ-like zinc finger domain-containing proteins, which share a characteristic tandemly arranged 4 × cysteine-rich signatures (CxxCxGxG) [4,7]. Although there has been no subsequent analysis on gametogenesis of the *eda3* mutant, recent studies have shown that PSA2 is a thylakoid protein that can bind to a PsaG-containing complex to regulate the assembly of photosystem I (PSI), and demonstrated that the *psa2* mutant has an abnormal assembly of grana thylakoids and poorly developed stroma thylakoids in chloroplasts [8,9]. 

In this study, we characterized the development of the female gametophyte of the *eda3* mutant and demonstrated the dual localization of EDA3 in both chloroplasts and the nucleus, in leaves and seeds, respectively.

## 2. Results and Discussion

**Figure 1:**
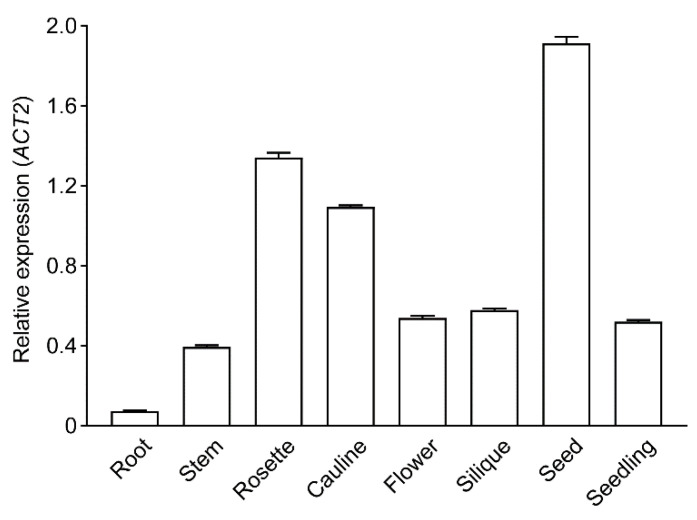
Expression pattern of *EDA3* in different tissues. Transcript abundances of *EDA3* in different tissues of *Arabidopsis thaliana* wild-type plants, together with the whole seedlings, were determined by qRT-PCR and normalized against the levels of *ACTIN2* (*ACT2*) (as a reference). Data represent means ± SEM (*n* = 3).

Although the *eda3* mutant was found to have arrested female gametophyte development, there has been no further characterization. In this study, we first compared the gene expression pattern of *EDA3*. Our qRT-PCR quantification showed that *EDA3* had the highest expression level in seeds, followed by rosette and cauline leaves (Figure 1). Its expression level in roots was the lowest among all different tissues analyzed (Figure 1). This suggested that *EDA3* might possess special functions while it is expressed in seeds.

**Figure 2:**
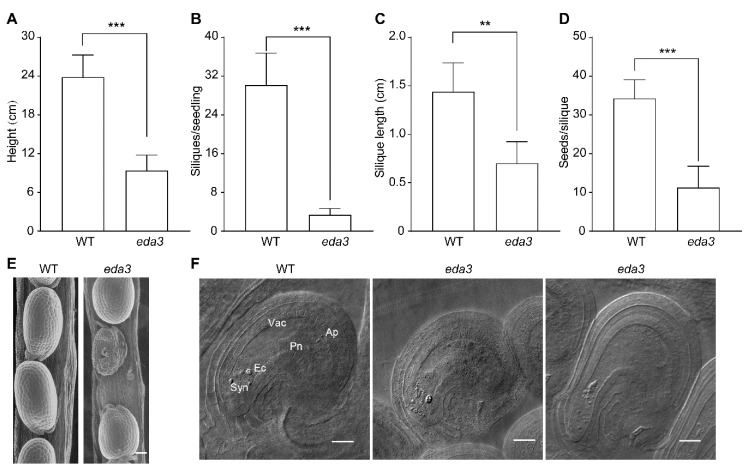
Phenotype of the *Arabidopsis thaliana eda3* mutant. (**A**) Heights of the seedlings; (**B**) Numbers of siliques per seedling; (**C**) Length of siliques; (**D**) Numbers of seeds per silique, were measured in the wild-type (WT) and *eda3* mutant seedlings. Seedlings for each line were cultivated under normal growth conditions. For (**A**,**B**), 6-week-old seedlings were measured. For (**C**,**D**), mature (brown) siliques were used for measurements. Data represent means ± SEM (*n* = 3). Asterisks indicate significant differences between the indicated lines (** *p* < 0.01, *** *p* < 0.001, Student’s *t*-test). (**E**) SEM observation of mature siliques of WT and *eda3*. Bar = 100 μm; (**F**) Microscopic observation of the embryo sacs of the WT and *eda3* plants. Vacuole (Vac), synergid (Syn), egg cell (Ec), polar nuclei (Pn), antipodal cells (Ap) are indicated. Bar = 20 μm.

Under normal growth conditions, seedlings of the *eda3* mutant were much smaller than the wild-type (WT) seedlings, demonstrating a pale-yellow pigmentation and dwarf phenotype (Figure S1) [8]. Our measurements showed that the *eda3* seedlings were significantly shorter than the WT seedlings (Figure 2A). This could be the result of the defective assembly of PSI and the impaired pigment biosynthesis, as reported earlier [8,9]. We further measured the length of siliques and also counted the silique and seed numbers. Our results indicated that, as compared with the WT seedlings, there were much fewer siliques in each *eda3* seedling (Figure 2B), and the siliques of the *eda3* mutant were also significantly shorter (Figure 2C). On average, there were 35.1 seeds in each WT silique, but the *eda3* plants had only 11.5 seeds per silique (Figure 2D). By dissecting the developing siliques, we observed both aborted and undeveloped ovules in the *eda3* siliques, which explained the lower seed number per silique in the mutant (Figure 2E and Table S1). Our microscopic observation clearly revealed that the *eda3* embryo sacs were arrested at the two-nuclear stage, whereas those of the WT developed normally (Figure 2F).

**Figure 3:**
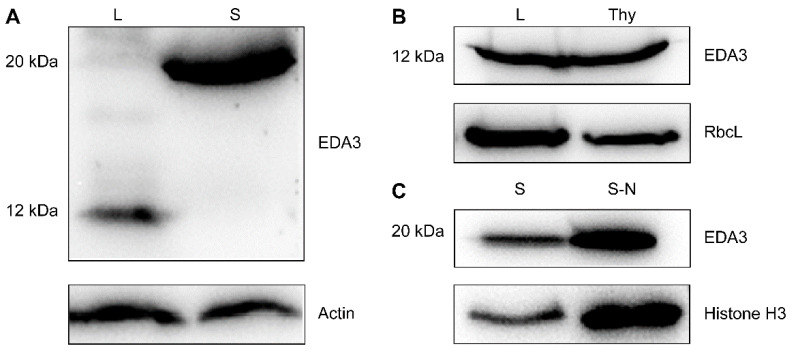
Immunoblot showing the dual localization of EDA3. (**A**) EDA3 localizes in leaves and seeds in the 12 and 20 kDa forms, corresponding to the truncated and full-length peptides, respectively. Actin was probed as a loading control; (**B**) EDA3 is a thylakoid protein in leaf cells. Rubisco large subunit (RbcL) was probed as a loading control; (**C**) EDA3 localizes in the nucleus in seeds. Histone H3 was probed as a loading control. L, leaves; S, seeds; Thy, thylakoid preparation; S-N, seed nuclear preparation.

Considering that EDA3 has previously been identified as a chloroplast protein, we analyzed its deduced amino acid for possible targeting signals. As expected, an N-terminal chloroplast transit peptide (cTP, Met^1^-Ser^75^) was found by the online software ChloroP (ver. 1.1, http://www.cbs.dtu.dk/services/ChloroP/, with a cleavage site score of 5.028). However, a nuclear localization signal (NLS, Asn^126^-Pro^151^) was also identified by cNLS Mapper (http://nls-mapper.iab.keio.ac.jp/, with a cut-off score set at 6.0 and searched the entire region) with a score of 6.7, suggesting a strong probability of an additional nuclear localization of EDA3 (Figure S2). This suggested that EDA3 might be truncated after being imported to chloroplast [10,11]. Calculated from the deduced amino acid, the relative molecular weights of the full-length and the truncated forms of EDA3 were 20 and 12 kDa, respectively. Then, we performed immunoblot analysis to compare proteins extracted from leaves and seeds using a specific antibody we raised against EDA3 (Figure S3). From our result, it was clear that a 12 kDa band corresponding to the truncated EDA3 was detected in the leaf sample, whereas a 20 kDa band corresponding to the full-size form was detected in the seed sample (Figure 3A). To further characterize the detailed localization, we isolated thylakoids from leaf cells and the nuclei from seeds. Our immunodetection demonstrated that the truncated EDA3 localized in the thylakoid fraction of leaf cells and the full-length protein had a nuclear localization in seeds (Figure 3B,C).

**Figure 4:**
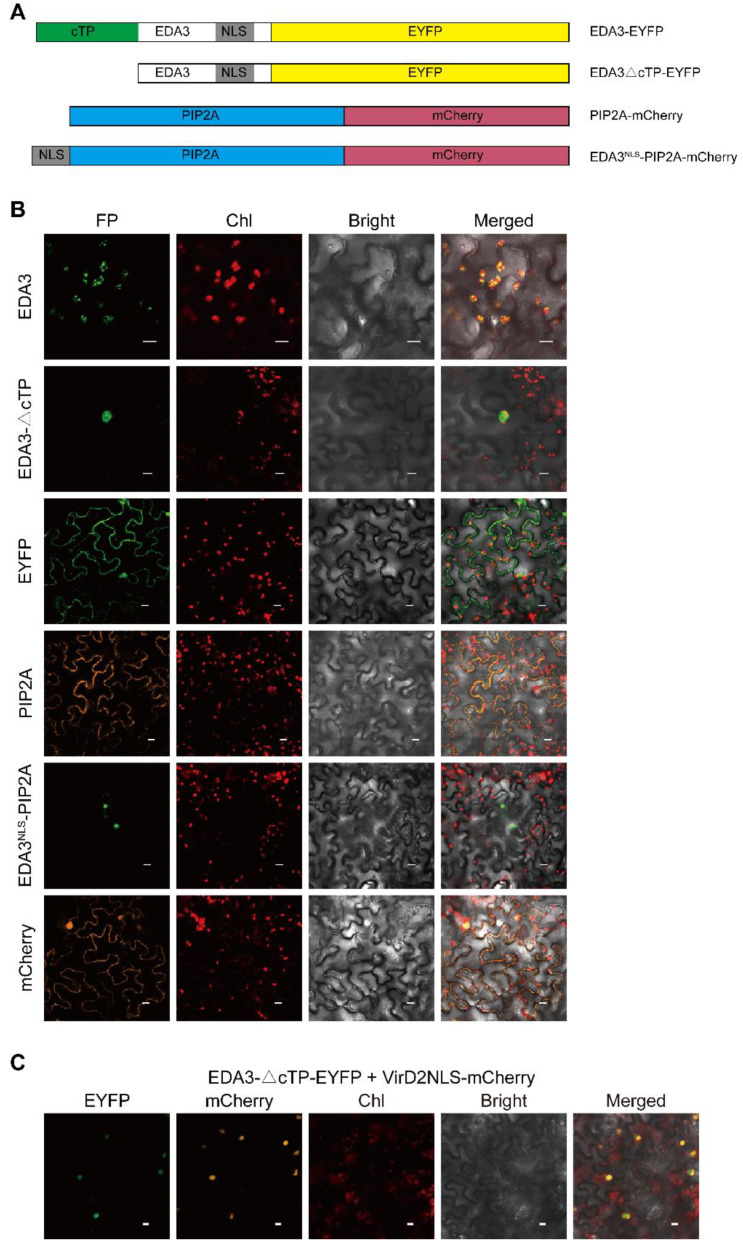
The nuclear localization signal (NLS) of EDA3 accounts for its nuclear targeting. (**A**) Schemes of the fusion proteins used in this assay. NLS, nuclear localization signal; cTP, chloroplast transit peptide; EDA3ΔcTP, truncated EDA3 with its cTP removed; EDA3^NLS^-PIP2A, PIP2A protein fused beyond the NLS of EDA3. The CaMV 35S promoter was used to drive the transient expression of each protein in tobacco leaves; (**B**) Transient expression EDA3 and EDA3ΔcTP fused upstream EYFP, and PIP2 and EDA3^NLS^-PIP2A fused upstream mCherry, in tobacco leaves by infiltration; (**C**) Co-localization of the EDA3ΔcTP-EYFP fusion protein with the nuclear localization marker protein VirD2NLS-mCherry. The excitation wavelengths for EYFP, mCherry, and chlorophyll auto-fluorescence were 488, 543, and 543 nm laser, respectively, and their corresponding recoding ranges were 500–530, 580–620, and 680–720 nm, respectively. Empty vectors expressing EYFP and mCherry were used as controls. FP, fluorescent protein. Bar = 10 μm.

To confirm the nuclear localization of EDA3, we transiently expressed its different fusion proteins in *N. benthamiana* leaves by infiltration (Figure 4A). A chloroplast localization was observed for the EDA3-EYFP fusion protein (Figure 4B). However, when the cTP was removed, the truncated EDA3 fused with EYFP (EDA3ΔcTP-EYFP) and demonstrated a nuclear localization. This suggested that the predicted NLS beyond cTP was able to target EDA3 into the nucleus. We further fused this NLS fragment and mCherry to the N- and C-termini of PIP2A, a protein marker for the plasma membrane, respectively, and expressed the protein in tobacco leaves [12]. Our observation indicated that the NLS was also able to deliver PIP2A into the nucleus (Figure 4B). In contrast, the full-length PIP2A alone was still localized to the plasma membrane in transfected tobacco leaf cells (Figure 4B). The VirD2NLS-mCherry fusion protein, of which mCherry is delivered to the nucleus by the fused NLS of the *Agrobacterium* VirD2 protein, has been accepted as a nuclear marker [13,14]. In this study, we also transiently co-expressed EDA3ΔcTP-EYFP with VirD2NLS-mCherry by infiltration. Our confocal observation clearly indicated a co-localization of these two fusion proteins in the nucleus (Figure 4C).

Taken together, our results collectively demonstrated that EDA3 had dual localization in chloroplasts and the nucleus. In addition to the previous reports which revealed its chloroplast localization and effects on PSI assembly, in this study, we identified its novel localization in the nucleus in seeds. It is not unusual for members of the DnaJ-like zinc finger domain-containing proteins to have different subcellular localizations with separate functions (Figure S4). For example, the ORANGE protein, which was originally identified to regulate chromoplasts biogenesis in non-pigmented plastids, also repressed the transcription factor TCP14 in the nucleus during de-etiolation (Figure S4) [7,14,15]. TsiP, which was associated with the chloroplast surface, was recruited to the nucleus for inducing the expression of stress-related genes [16]. Moreover, EDA3 is not the only member of this protein family involved in both chloroplast functions and embryo development. Gene silencing mutants of ANGULATA 7 also exhibit defective thylakoid membrane arrangement and are embryo lethal (Figure S4) [2,3,17].

However, it is still unclear how EDA3 functions during the development of the female gametophyte in *A. thaliana*. From our results, we were unable to rule out the possibility that such defective development was a result of the impaired photosynthetic capability, which might affect specific metabolic pathways that are crucial for embryo development [2,3,8]. It would be interesting to elucidate how such a dual localization is manipulated during development, and whether or not other members of the DnaJ-like zinc finger domain-containing protein family also possess nuclear localization.

## 3. Materials and Methods

### 3.1. Plant Materials and Growth Conditions

All *Arabidopsis thaliana* lines used in this study were in Col-0 wild-type (WT) background. The *eda3* mutant seeds (CS445540, with the T-DNA insertion at the 5’-UTR) were purchased from the *Arabidopsis* Biological Resource Center (ABRC, Ohio State Univ., Columbus, OH, USA) [8]. Seeds were surface sterilized, and then plated onto Murashige and Skoog (MS) medium containing 2% sucrose and 0.8% agar. After stratification for 3 d, at 4 ℃, in the dark, seeds were germinated at 22 ℃ under a light intensity of 120 µmol photons m^−2^ s^−1^ with a 14 h/10 h light/dark regime. Two-week-old seedlings were moved to grow in a mixture of peat moss, vermiculite, and perlite (at 1:1:1) under the same conditions [18].

### 3.2. RNA Extraction, Reverse Transcription, and Gene Expression Analysis 

Total RNA was extracted using the RNAiso reagent (TaKaRa, Shiga, Japan), and reverse transcribed into cDNA with a PrimeScript Double Strand cDNA Synthesis Kit (TaKaRa), following the manufacturer’s instructions. Gene expression levels were quantified by quantitative real-time PCR (qRT-PCR) using SYBR Premix ExTaq II (TaKaRa) with a Thermal Cycler Dice Real-Time System TP800 (TaKaRa). Transcript abundances were calculated according to the comparative *C_T_* method [19]. For each sample, at least three biological replicates were analyzed, and each with three repeats. *ACT2* was used as a reference for normalizing gene expression. All primers used in this study are listed in Supplemental Table S2.

### 3.3. Protein Extraction, Antiserum Preparation, and Immunoblot Analysis 

Total proteins were extracted from rosette leaves or seeds using the trichloroacetic acid (TCA) precipitation method [18]. Approximately 200 mg of rosette leaves or 30 mg seeds were first homogenized in liquid nitrogen before 500 μL cold 10% TCA in acetone was added and mixed thoroughly by a vigorous vortex. The mixture was incubated at −20 ℃ for at least 2 h, and then centrifuged at 4 ℃, 15,000× *g* for 10 min. The pellet was washed several times with cold acetone, air dried briefly, and solubilized in 9 M urea (in 100 mM phosphate buffered saline) with sonication. After centrifugation at 4 ℃, 15,000× *g* for 10 min, the supernatant was used for analysis. Protein samples were mixed with equal amounts of 2 × SDS loading buffer, separated by 15% SDS-polyacrylamide gel electrophoresis (PAGE) and, subsequently, blotted onto a nitrocellulose membrane (GE Healthcare, Chicago, Illinois, USA) for immunodetection. 

Nuclear proteins were extracted from seeds, according to Xu et al. [20]. About 200 mg seeds were frozen in liquid nitrogen, ground to a fine powder, and homogenized in lysis buffer (20 mM Tris-HCl, pH 7.4, 25% glycerol, 20 mM KCl, 2 mM EDTA, 2.5 mM MgCl_2_, 250 mM sucrose, 1 mM DTT, and 1 mM PMSF) at 4 ℃. The homogenate was filtered through four layers of miracloth (Merck Millipore, Darmstadt, Germany) [15]. The nuclei were pelleted by centrifugation at 1500× *g* for 10 min, washed three times with nuclei resuspension buffer (20 mM Tris-HCl, pH 7.4, 25 % glycerol, 2.5 mM MgCl_2_, 0.2% Triton X-100) at 4 ℃, resuspended in 2 mL ice-cold nuclei resuspension buffer (20 mM Tris-HCl, pH 7.4, 25% glycerol, 2.5 mM MgCl_2_), and then centrifuged at 1500× *g* and 4 °C for 10 min. The pelleted nuclei were suspended in 1 × SDS loading buffer, heated at 95 ℃ for 10 min, and then subjected to SDS-PAGE analysis and immunodetection. 

For thylakoid proteins, about 10 g of rosette leaves were homogenized briefly in cold grinding buffer (50 mM HEPES-KOH, pH 7.5, 330 mM sorbitol, 2 mM EDTA, 1 mM MgCl_2_, 5 mM ascorbate, 0.05% bovine serum albumin, 10 mM NaF, 1 mM PMSF), and then filtered through two layers of miracloth. After being centrifuged at 4 ℃, 2500× *g* for 5 min, the pelleted chloroplasts were resuspended in shock buffer (50 mM HEPES-KOH, pH 7.5, 5 mM sorbitol, 5 mM MgCl_2_, 10 mM NaF, 1 mM PMSF) and lysed by resting on ice for 10 min. Broken chloroplasts were pelleted by centrifugation at 4 ℃, 2500× *g* for 5 min, washed twice with storage buffer (50 mM HEPES-KOH, pH 7.5, 100 mM sorbitol, 10 mM MgCl_2_, 10 mM NaF, 1 mM PMSF), and then centrifuge at 4 ℃, 2500× *g* for 5 min, to pellet the thylakoids [21]. Pelleted thylakoids were mixed with 1 × SDS loading buffer, heated at 95 ℃ for 10 min, and then subjected to SDS-PAGE analysis and immunodetection. 

A peptide (GLPNNKGLLRRPGA) corresponding to Gly^157^ to Ala^170^ of EDA3 was synthesized and used as the antigen to immune rabbits by GenScript (Nanjing, China) (Figure S2). The antibody against ACTIN was purchased from Sangon (Shanghai, China), and antibodies against histone 3 and Rubisco large subunit (RbcL) were purchased from Beyotime (Shanghai, China). Horseradish peroxidase (HRP)-conjugated secondary antibody against rabbit IgG was from Sangon (Shanghai, China). Common protocols and the manufacturers’ manuals for SDS-PAGE, blotting, and immunodetection using the BeyoECL Star Western Blotting Substrate (Beyotime) were followed [22].

### 3.4. Subcellular Localization Assay

For subcellular localization analysis, we first predicted the N-terminal chloroplast transit peptide (cTP) of EDA3 using TargetP (version 2.0, http://www.cbs.dtu.dk/services/TargetP) and ChloroP (version 1.1, http://www.cbs.dtu.dk/services/ChloroP) [23,24], and the nuclear localization signal using cNLS Mapper (http://nls-mapper.iab.keio.ac.jp/) [25] (Figure S2). A full-length open reading frame (ORF) of *EDA3* was amplified from the 1st strand cDNA pool using the primer pair EDA3-HF and EDA3-ER, and subsequently cloned into the NcoI site of pCNHP-EYFP, which we constructed based on pCAMBIA1300 and harbors sequentially the enhanced Cauliflower mosaic virus (CaMV) 35S promoter, synthetic 5′ and 3′ untranslated regions of Cowpea mosaic virus *RNA2* flanking the coding region fused in frame to the 5′-end of the gene for either enhanced yellow fluorescent protein (EYFP) or mCherry, and the Heat Shock Protein (HSP) terminator from *A. thaliana*, to generate 35S:EDA3-EYFP [26]. To amplify the coding region for EDA3 with its cTP truncated, the primer pair EDA3-TrunF and EDA3-ER was used. The amplicon was cloned into pCNHP-EYFP to generate 35S:EDA3-ΔcTP-EYFP. We also brought the coding region for the predicted NLS of EDA3 to the 5′ end of the full-length ORF of *PIP2A*, which encodes a plasma membrane protein [12], by sequential PCR using NLS-PIP2A-EYFP-F1 to NLS-PIP2A-EYFP-F4 with the reverse primer PIP2A-ER. The final amplicon was cloned into pCHNP-mCherry. Full-length ORF of PIP2A was amplified using the primer pair PIP2A-FHF and PAP2A-ER, and the amplicon was also cloned into pCHNP-mCherry. We also further cloned the 35S:EDA3-ΔcTP-EYFP cassette into the binary vector pPZP-RCS2-Bar (ABRC), together with the cassette from pSAT6-mCherry-VirD2NLS (ABRC), to simultaneously express both EDA3-ΔcTP-EYFP and the nuclear marker protein VirD2NLS-mCherry in tobacco leaves [13,14]. The transformation and cultivation of *Agrobacterium tumefaciens* strain GV3101 and the infiltration of *Nicotiana benthamiana* leaves were performed, as described [26,27].

For all PCR amplifications, high-fidelity PrimeSTAR HS DNA polymerase (TaKaRa) was used, according to the manufacturer’s instruction. After electrophoresis, the amplicon was purified from the gel using a SanPrep Column DNA Gel Extraction Kit (Sangon, Shanghai, China). 

### 3.5. Microscopic Observations

*Arabidopsis siliques* were observed under a Quanta 250 FEG scanning electron microscope (Thermo Fisher, Hillsboro, OR, USA). For the observation of the embryo sac, flower buds were sequentially fixed twice in methanol (5 min each), three times in ethanol (5 min each), and then in Hoyer’s solution for at least 2 h [28]. Pistils were dissected and observed as described using a FLUOVIEW FV1000 Laser Confocal Microscopy System (Olympus, Tokyo, Japan) [4]. The fluorescence signal of infiltrated tobacco leaves was detected using the same confocal system. The EYFP fluorescent was excited with 488 nm laser and the emitted light was recorded from 500 to 530 nm laser. The mCherry fluorescent was excited with 543 nm laser, recorded from 580 to 620 nm. The 543 nm laser excitation and 680 to 720 nm recording range were used for chlorophyll autofluorescence observation [18].

### 3.6. Statistical Analysis

Statistical significance was tested using GraphPad Prism 6 (GraphPad Software, San Diego, CA, USA). To determine statistical significance, we employed Student’s *t*-test. Differences were considered to be significant at *p* < 0.05.

## Supplementary Materials

Supplementary materials can be found at https://www.mdpi.com/1422-0067/21/21/7979/s1, Table S1: Seed development in the wild-type and *eda3* mutant plants, Table S2: Primers used in this study, Figure S1: Phenotype of the *eda3* mutant, Figure S2: Deduced amino acid sequence of EDA3, Figure S3: Validation of the antibody against EDA3, Figure S4: Sequences of EDA3, ORANGE, TsiP1, and ANGULATA 7.

## Abbreviations

NLS	Nuclear localization signal
cTP	Chloroplast transit peptide
EYFP	Enhanced fluorescent protein
EDA	Embryo sac development arrest
