# Supplementary material for: The Nuclear Localization of the DnaJ-Like Zinc Finger Domain-Containing Protein EDA3 Affects Seed Development in Arabidopsis thaliana
Source: Int J Mol Sci. 2020 Oct 27;21(21):7979. doi: 10.3390/ijms21217979 (PMC7662858; doi:10.3390/ijms21217979)
Supplement: Supplementary file 1 [file ijms-21-07979-s001.pdf]

Table S1. Seed development in the wild-type and *eda3* mutant plants.

| | Aberrant seeds | | Total seeds | Percentage of aberrant seeds |
|---|---|---|---|---|
| | Undeveloped ovules | Aborted seeds | | |
| wild type | 11 | 4 | 962 | 1.6% |
| *eda3* | 363 | 79 | 998 | 44.3% |

Table S2. Primers used in this study.

| Primer name | Sequence (5'-3') |
|---|---|
| For qRT-PCR | |
| EDA3-QF | CTTCCTCAGAGACTACCCACAAAG |
| EDA3-QR | GAACCTCCTGGTTTCTTGTCTAAAGC |
| ACT2-QF | CCAACATATGCATCCTTCTGGTTCATCCCA |
| ACT2-QR | TGGCTGAGGCTGATGATATTCAACCAATCG |
| For expressing EDA3-EYFP | |
| EDA3-HF | TCGAATTCCTGCAGCCCGGGATGGCGGCATCATCATCTCATCTCT |
| EDA3-ER | CCCTTGCTCACCATACTAGTTGAATCAGGAAGAAGCCGACCATTG |
| For expressing EDA3ΔcTP-EYFP | |
| EDA3-TrunF | TCGAATTCCTGCAGCCCGGGATGGCAGCTTTAATTAGCAATTCTT |
| For expressing PIP2A-mCherry | |
| PIP2A-HF | TCGAATTCCTGCAGCCCGGGATGGCAAAGGATGTGGAAGCCGTTC |
| PIP2A-ER | CCCTTGCTCACCATACTAGTGACGTTGGCAGCACTTCTGAATGAT |
| For expressing PIP2A fused with the NLS of EDA3 and mCherry | |
| NLS-PIP2A-EYFP-F1 | CTTCTTAGAAGGCCTGGTGCTCGTGAGCTAGCAAAGGATGTGGAAGCCGT |
| NLS-PIP2A-EYFP-F2 | TTAGGTACAGGTTTACCAAACAACAAAGGCCTTCTTAGAAGGCCTGGTGC |
| NLS-PIP2A-EYFP-F3 | GGTCGAGGTAAGCTTGTTTGTCCGGTCTGTTTAGGTACAGGTTTACCAAA |
| NLS-PIP2A-EYFP-F4 | TCGAATTCCTGCAGCCCGGGATGGGTCGAGGTAAGCTTGTTTGTC |

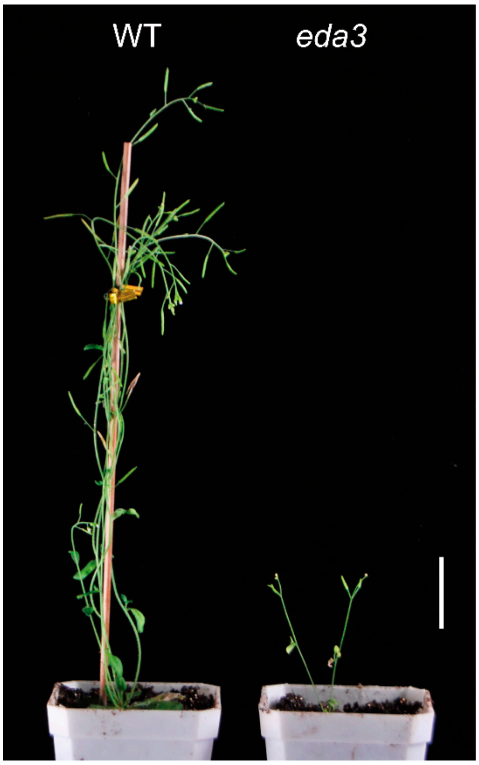


Figure S1. The *eda3* mutant shows a retarded growth phenotype under normal conditions.

Bar = 3 cm.

MAASSSHLFALPSPASPFLSAPNRNRVRVLAKSCPENQSFDSNDSDSSSE 50

TTHKAQGDQKSVSRRQWMTACVCASAALISNSYTFVSVQSAAALDKKPGG 100

SCRNCQGSGAVLCDMCGGTGKWKAL*NRKRAKDVYEFTECPNCYGRGKLVC* 150

*P*VCLGT**GLPNNKGLLRRPGA**RELLEKMYNGRLLPDS 186

Figure S2. Deduced amino acid sequence of EDA3.

Predicted chloroplast transit peptide is underlined. The nuclear localization signal is in italic. The peptide used as the antigen for raising antibody against EDA3 is in boldface.

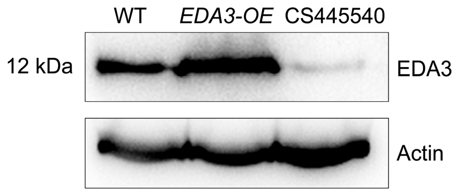


Figure S3. Validation of the antibody against EDA3.

Seedlings of the wild-type (WT), *EDA3*-overexpression (*EDA3-OE*), and the T-DNA insertion mutant (CS445540) were grown under normal conditions. Leaves from different lines were harvested and used for immunoblot analysis using the antibody we raised against EDA3. Actin was probed as a reference.

>EDA3 (AT2G34860)
MAASSSHLFALPSPASPFLSAPNRNRVRVLAKSCPENQSFDSNDSDSSSE
TTHKAQGDQKSVSRRQWMTACVCASAALISNSYTFVSVQSAAALDKKPGG
SCRNCQGSGAVLCDMCGGTGKWKALNRKRAKDVYEFTECPNCYGRGKLVC
PVCLGTGLPNNKGLLRRPGARELLEKMYNGRLLPDS

>ORANGE (AT5G61670)
MSSLGRILSVSYPPDPYTWRFSQYKLSSSLGRNRRLRWRFTALDPESSSL
DSESSADKFASGFCIIEGPETVQDFAKMQLQEIQDNIRSRRNKIFLHMEE
VRRLRIQQRIKNTELGIINEEQEHELPNFPSFIPFLPPLTAANLKVYYAT
CFSLIAGIILFGGLLAPTLELKLGIGGTSYADFIQSLHLPMQLSQVDPIV
ASFSGGAVGVISALMVVEVNNVKQQEHKRCKYCLGTGYLACARCSSTGAL
VLTEPVSAIAGGNHSLSPPKTERCSNCSGAGKVMCPTCLCTGMAMASEHD
PRIDPFD

>TsiP1 (AAD18030)
MASSSTCTCSCRPIITAKSNIINRFVTPRGIQLIFHGNPRLKQVPRIFAV
RASAVDSSSSFVERMEKAWLISKQPRPIVCSTCGSNGHVECKWCSGTGFF
VLGDNMLCQVPSRNTSCVICAGKGSVCCTDCKGTGHRAKWLGEPPLPNPP
IAKE

>ANGULATA7 (AT5G53860)
MSRGPGRLIQNVTQFADAQFKQFSTRYGQQVIDILDFPIKLVLSPFTLAF
DIAGSAPRGFGIPEFISKISYLSVFAVATLGTYDIALDLGKKVICQRDCK
TCNGWQALRCTMCKGTGSVHYQIKDYNLRSGEKPTADCVADAIVENRAEL
VHLPSSFNHSAPLPSKDCPTCDGTGAMSCTECKNKLQVRISADDIMEPPW
KAYNVLKKMDYPYEHIVHSMKDPSIANFWLITLPQIVGGFDYDEDVKKKI
WWQYELNLLNPHLGWFCYTNIVSIPYLDRFVLWNWRHLWDTDESMRYDQL
RDLVAKRNPGWEYLQDALVSIDPVRAREDPVIVKNVPYYKAKKSLEAEVT
KLNPPPRPQNWGELNLPLNISSWSEEDLKNPAKLYEKTVLLNAQREIADK
ILDAQWEAKWRQEKVEEMLEQKVRPYIQDSSMAVLPQPILLKSQKKAQKG
SRQRKWWFF

Figure S4. Sequences of EDA3, ORANGE, TsiP1, and ANGULATA 7.

Conserved cysteine-rich DnaJ-like zinc finger domains are underlined.
